# Competing Risk Bias in Prognostic Models Predicting Hepatocellular Carcinoma Occurrence: Impact on Clinical Decision-making

**DOI:** 10.1016/j.gastha.2021.11.008

**Published:** 2022-02-03

**Authors:** Hamish Innes, Philip Johnson, Scott A. McDonald, Victoria Hamill, Alan Yeung, John F. Dillon, Peter C. Hayes, April Went, Stephen T. Barclay, Andrew Fraser, Andrew Bathgate, David J. Goldberg, Sharon J. Hutchinson

**Affiliations:** 1 School of Health and Life Sciences, Glasgow Caledonian University, Glasgow, UK; 2 Public Health Scotland, Glasgow, UK; 3 Division of Epidemiology and Public Health, University of Nottingham, Nottingham, UK; 4 Department of Molecular and Clinical Cancer Medicine, University of Liverpool, Liverpool, UK; 5 Division of Molecular and Clinical Medicine, School of Medicine, University of Dundee, Dundee, UK; 6 Royal Infirmary of Edinburgh, Edinburgh, UK; 7 Glasgow Royal Infirmary, Glasgow, UK; 8 Aberdeen Royal Infirmary, Aberdeen, UK; 9 Queen Elizabeth University Hospital, Glasgow, UK

**Keywords:** Prognosis, Liver Cancer, Risk Stratification, Fine-Gray, Competing Risks

## Abstract

**Background and Aims:**

Existing models predicting hepatocellular carcinoma (HCC) occurrence do not account for competing risk events and, thus, may overestimate the probability of HCC. Our goal was to quantify this bias for patients with cirrhosis and cured hepatitis C.

**Methods:**

We analyzed a nationwide cohort of patients with cirrhosis and cured hepatitis C infection from Scotland. Two HCC prognostic models were developed: (1) a Cox regression model ignoring competing risk events and (2) a Fine-Gray regression model accounting for non-HCC mortality as a competing risk. Both models included the same set of prognostic factors used by previously developed HCC prognostic models. Two predictions were calculated for each patient: first, the 3-year probability of HCC predicted by model 1 and second, the 3-year probability of HCC predicted by model 2.

**Results:**

The study population comprised 1629 patients with cirrhosis and cured HCV, followed for 3.8 years on average. A total of 82 incident HCC events and 159 competing risk events (ie, non-HCC deaths) were observed. The mean predicted 3-year probability of HCC was 3.37% for model 1 (Cox) and 3.24% for model 2 (Fine-Gray). For most patients (76%), the difference in the 3-year probability of HCC predicted by model 1 and model 2 was minimal (ie, within 0 to ±0.3%). A total of 2.6% of patients had a large discrepancy exceeding 2%; however, these were all patients with a 3-year probability exceeding >5% in both models.

**Conclusion:**

Prognostic models that ignore competing risks do overestimate the future probability of developing HCC. However, the degree of overestimation—and the way it is patterned—means that the impact on HCC screening decisions is likely to be modest.

## Introduction

Hepatocellular carcinoma (HCC) is a leading cause of cancer mortality, responsible for ∼800,000 deaths every year.^1^ Most HCCs occur in patients who have cirrhosis of the liver. Thus, clinical guidelines recommend that all patients with cirrhosis should receive biannual ultrasound screening.^2^^,^^3^ The goal of screening is to increase detection of HCC at an early stage, where it is potentially curable via therapies such as hepatic resection, ablation, and transplantation. Unfortunately, abdominal ultrasound screening is inherently insensitive and tends to be poorly implemented in Western settings; as such HCC is usually detected too late for curative treatments to be given.^4^, ^5^, ^6^

There is an increasing appetite for a more targeted approach to HCC surveillance, where screening resources are focused on patients with cirrhosis who stand to gain the most from active surveillance.^7^, ^8^ This approach hinges on the availability of appropriate risk-stratification tools. Accordingly, the number of models predicting a patient’s risk of HCC occurrence in a surveillance context has increased dramatically over the last 5 years.^9^, ^10^, ^11^, ^12^, ^13^, ^14^, ^15^, ^16^ However, such models all share one potentially serious limitation: namely, the failure to take the competing risk event of non-HCC mortality into account. This is potentially a serious limitation because typically, patients with cirrhosis do not just face a high risk of HCC, but a high risk of dying from non-HCC–related causes too. The high risk of non-HCC mortality in patients with cirrhosis relates mainly to death from decompensated cirrhosis,^17^ but other causes may contribute too, such as extrahepatic cancers.^18^ Thus, even though a patient may have a high risk of HCC incidence, if they are also at very high risk of non-HCC mortality, then the actual probability of developing HCC in the next 3 years will be mitigated substantially (ie, because there is a strong chance that the patient will die first from causes unrelated to HCC).^19^^,^^20^ Failing to take this into account will lead to inflated estimates of the future probability for HCC. This is generally known as “competing risk bias”.^21^

Until now, the impact of competing risk bias in a HCC prediction modeling context has not been assessed empirically. This means we do not know either the extent to which existing models are biased^9^, ^10^, ^11^, ^12^, ^13^, ^14^, ^15^, ^16^ or what the potential knock-on effects may be for clinical decision-making. Using data from a Scottish cohort of patients with cirrhosis and cured hepatitis C infection, the goal of this study was to quantify competing risk bias in a HCC prediction modeling context.

## Methods

### Data Sources

The Scottish HCV clinical database has been described extensively elsewhere.^22^ In brief, it is a retrospective cohort study >25,000 patients in Scotland who have attended a specialist liver clinic appointment for care/management of chronic HCV infection. The database records information collected during routine clinical care such as details of antiviral treatment episodes and the results of laboratory tests. It is also linked routinely to national health registries in Scotland, including the in-patient hospitalization (SMR01), cancer (SMR06), and mortality registers.

### Study Population

We included all patients with cirrhosis before initiating antiviral therapy and who subsequently achieved sustained viral response (SVR). All SVRs were included irrespective of the antiviral treatment regimen. If a patient had more than one treatment episode resulting in SVR, then the first episode was selected. Individuals with a diagnosis of HCC before SVR achievement were excluded. We also excluded participants who were missing data for selected prognostic factors.

Liver cirrhosis was defined as compensated or decompensated cirrhosis diagnosed during routine clinical investigation. Diagnoses were typically made following liver biopsy, transient elastography, abdominal ultrasound, clinical examination, and routine liver function tests, as per clinical guidelines at that time.

### Primary Outcome Event

The primary outcome event was an incident HCC diagnosis, defined as a first-time ICD10:C22.0 code in the (a) SMR06 Scottish cancer registry or (b) the SMR01 Scottish hospital admission registry or (c) Scottish mortality register. This HCC case definition has been internally validated in previous work—for example, by assessing agreement between HCC recorded in a hospital admission context and HCC recorded subsequently as a cause of death.^23^ In the present study, the C22.0 code was only used to define HCC if it appeared in the principal diagnostic/cause of death position, to minimize misclassification. The earliest HCC diagnosis/presentation date across these 3 registries was assumed to be the HCC incidence date.

### Prognostic Factors

To ensure generalizability, our aim was to select prognostic factors that are being used in existing prediction models for HCC occurrence in a surveillance context. To this end, we reviewed 8 models recently published in leading liver disease journals^9^, ^10^, ^11^, ^12^, ^13^, ^14^, ^15^, ^16^; prognostic factors included in more than one model were selected (Table A1).

Some prognostic factors for HCC are dynamic insofar as they fluctuate over time. To be consistent with previous studies,^12^ prognostic factors derived from laboratory tests (ie, albumin, platelet count, etc.) were calculated from the most recent test on or before the start of antiviral treatment. Tests conducted more than twelve months before initiating treatment were excluded. All other prognostic factors relate specifically to the time of SVR achievement.

### Statistical Analysis

#### Definition of Risk Sets

All statistical analyses were underpinned by survival analysis methods. Follow-up time began at the date of SVR achievement. This was defined as 6 months after the treatment completion date for episodes initiated before the year 2014 (ie, SVR24) and 3 months after the treatment completion date for episodes initiated from 2014 onward (ie, SVR12). This aligns with how SVR was defined by clinicians during the time period of this study. Follow-up ended at the date of incident HCC (if at all), mortality (if at all), or the date of study completion. The study completion date was January 1, 2020, corresponding to the date the hospital admission register was complete to at the time of analysis.

#### Model 1 and Model 2 Development

We developed models predicting the risk of HCC occurrence—model 1 and model 2.

Both models were developed from the same data set and included the same set of predefined prognostic factors.

The only difference between these 2 models related to the treatment of non-HCC mortality as a competing risk event.

Model 1 was derived from a standard Cox regression model, ignoring non-HCC mortality as a competing risk.

Conversely, model 2 was derived from Fine-Gray regression,^24^ which models the cumulative incidence of HCC directly. In this analysis, rather than being censored, individuals who die of non-HCC mortality before developing HCC are effectively kept in the risk set until the study completion date.^25^

The same set of prognostic factors was included in both models regardless of the statistical significance of their association with HCC.

The assumption of proportional hazards was verified using the Schoenfeld residual test. This test was applied to both the HCC cause-specific model (ie, model 1) and the equivalent cause-specific model for non-HCC mortality (ie, the competing risk event).

#### Discrimination

First, we assessed the discriminative ability of both models using Harrel’s C-index and the Wolbers modified C-index, where the latter accounts for competing risk.^26^

Moreover, we assessed the agreement between being “high risk” for model 1 and being “high risk” for model 2. Vice versa, we assessed the overlap between being “low risk” for model 1 and “low risk” for model 2. At present, there is no clinical consensus regarding the definition of “high” HCC risk for patients with cirrhosis. Thus, in this analysis, high risk was defined arbitrarily as a risk score in the 67th percentile or greater; low risk was defined as a risk score in the 33rd percentile or lower. Alternative definitions were considered in sensitivity analyses.

#### Generating Three-year Predicted Probability of HCC

For model 1, the 3-year absolute probability of HCC was calculated using the equation:

$$1-{\text{S}}_{\text{0}}{\left(\text{t}\right)}^{\exp\left(\text{linear}\;\text{predictor}\right)}$$

Where t = 3 years, and S_0_(t) refers to the estimated 3-year HCC-free survival for individuals with zero for all independent variables in the model.

For model 2, the 3-year probability of HCC was calculated using the analogous equation for Fine-Gray regression:

$$1-{\text{CIF}}_{\text{0}}{\left(\text{t}\right)}^{\exp\left(\text{linear}\;\text{predictor}\right)}$$

Where t = 3 years, and CIF_0_(t) refers to the predicted cumulative incidence at 3 years for individuals with zero for all independent variables in the model.

The agreement between the 3-year risk of HCC in model 1 and model 2 was assessed using a Bland-Altman plot,^27^ histograms, and descriptive statistics.

All authors had access to the study data and reviewed and approved the final article. All statistical analyses were performed in Stata, version 17.^28^

## Results

### Derivation of Final Sample Size

A total of 2245 patients met our inclusion criteria from the Scottish cohort. We then excluded 106 patients with HCC before treatment completion. Five hundred ten participants were then excluded because they were missing data for one or more selected prognostic factors. Thus, the final sample size was 1629 (Figure A1).

### Characteristics of the Final Sample

Patients were mainly middle-aged (ie, between 40 and 65 years old), were male (>70%) of white ethnicity (>90%), and had a history of injecting drug use (77.1%). Most patients had achieved SVR via an interferon-free regimen (60.2%). About one patient in 10 had decompensated cirrhosis at the time of achieving SVR (10.6%) (Table 1).

**Table 1:** Description of the Final Cohort (N = 1629)

Characteristic	W/o HCC		With HCC		All patients	
	Estimate	n	Estimate	n	Estimate	N
Demographic factors						
Mean age, years	49.9 (sd: 8.6)	1547	54.8 (sd: 7.1)	82	50.1 (sd: 8.6)	1629
% male gender	73.4%	1547	91.5%	82	74.3%	1629
% white ethnicity	93.8%	1547	97.6%	82	94.0%	1629
Clinical factors						
% IFN-free therapy	60.8%	1547	48.8%	82	60.2%	1629
% decompensated cirrhosis	10.1%	1547	19.5%	82	10.6%	1629
% past genotype 3 infection	50.4%	1537	61.0%	82	50.9%	1619
% IDU history	77.5%	1278	69.6%	69	77.1%	1347
Laboratory markers						
Mean platelet count, 109 cells/L	150.9 (sd: 67.3)	1547	112.2 (sd: 53.9)	82	150.0 (sd: 67.2)	1629
Mean ALT, IU/L	87.1 (sd: 70.3)	1547	91.3 (sd: 84.7)	82	87.3 (sd: 71.1)	1629
Mean AST, IU/L	84.0 (sd: 56.7)	1547	91.2 (sd: 64.1)	82	84.4 (sd: 57.1)	1629
Mean albumin, g/L	37.1 (sd: 5.2)	1547	35.2 (sd: 4.7)	82	37.0 (sd: 5.2)	1629

Laboratory markers are based on values at the time of treatment initiation, whereas all other dynamic variables (eg, age) are based on the value at SVR achievement. See main text for further explanation.

ALT, alanine aminotransferase; AST, aspartate aminotransferase; IDU, intravenous drug use; IFN, interferon.

The characteristics of patients excluded from the final sample (ie, N = 510) were similar to those who were included (see Table A2).

### Cumulative Incidence of HCC and Non-HCC Mortality

Patients were followed for a mean 3.9 years after SVR achievement. During this follow-up period, 82 incident HCC events and 159 non-HCC–related deaths occurred. The cumulative incidence of HCC and non-HCC mortality was 3.4% (95% confidence interval [CI]: 2.7–4.3) and 8.6% (95% CI: 6.3–8.8), respectively (Table A3).

### Prognostic Factor Selection

Of the prognostic models reviewed,^9^, ^10^, ^11^, ^12^, ^13^, ^14^, ^15^, ^16^ we identified 9 prognostic factors that were included in at least 2 models (Table A2). These were as follows: alanine aminotransferase, aspartate aminotransferase, age, albumin, gender, platelet count, liver stiffness, SVR achievement, and gamma glutamyl transferase (GGT). Liver stiffness and GGT were excluded because data for these prognostic factors were not available in our UK data sets. SVR achievement was excluded as a prognostic factor because all patients in our UK data sets had achieved SVR. Thus, the remaining 6 prognostic factors were included in our model development process.

### Prognostic Factor–HCC Association

Of the 6 prognostic factors selected, 4 were associated with an increased risk of incident HCC: lower platelet count, older age, male gender, and lower albumin. Conversely, aspartate aminotransferase and alanine aminotransferase were not associated with HCC in either model.

The biggest difference between model 1 and model 2 related to the effect size for albumin, which was stronger in model 1 (hazard ratio [HR]: 0.75; 95% CI: 0.60–0.93; *P* = .010) than in model 2 (sub distribution HR [sdHR]: 0.82; 95% CI: 0.67–1.01; *P* = .064). However, the difference was not statistically significant. Otherwise, the effect sizes for each prognostic factor were similar between the 2 models (Table 2). No significant violations in the proportional hazard assumption were observed (Table A4).

**Table 2:** Regression Coefficients for Cox and Fine-Gray Models

Covariate	Model 1 (Cox)		Model 2 (Fine-Gray)	
	HR (95% CI)	*P*-value	sdHR (95% CI)	*P*-value
Platelet count, per 10-unit increase	0.91 (0.87–0.95)	2.8 × 10^−5^	0.91 (0.87–0.95)	5.6 × 10^−5^
Age, per 1-y increase	1.07 (1.05–1.10)	2.9 × 10^−9^	1.07 (1.05–1.09)	1.4 × 10^−12^
Male gender^a^	4.60 (2.11–10.06)	.0001	4.53 (2.05–10.03)	.0002
Albumin, per 5-unit increase	0.75 (0.60–0.93)	.01	0.82 (0.67–1.01)	.064
ALT	0.99 (0.94–1.04)	.94	0.99 (0.94–1.05)	.74
AST	1.00 (0.93–1.07)	.93	1.00 (0.94–1.07)	.91

ALT, alanine aminotransferase; AST, aspartate aminotransferase; HR, hazard ratio.

a 7 of 82 HCC cases are female.

### Discrimination

**Figure 1:**
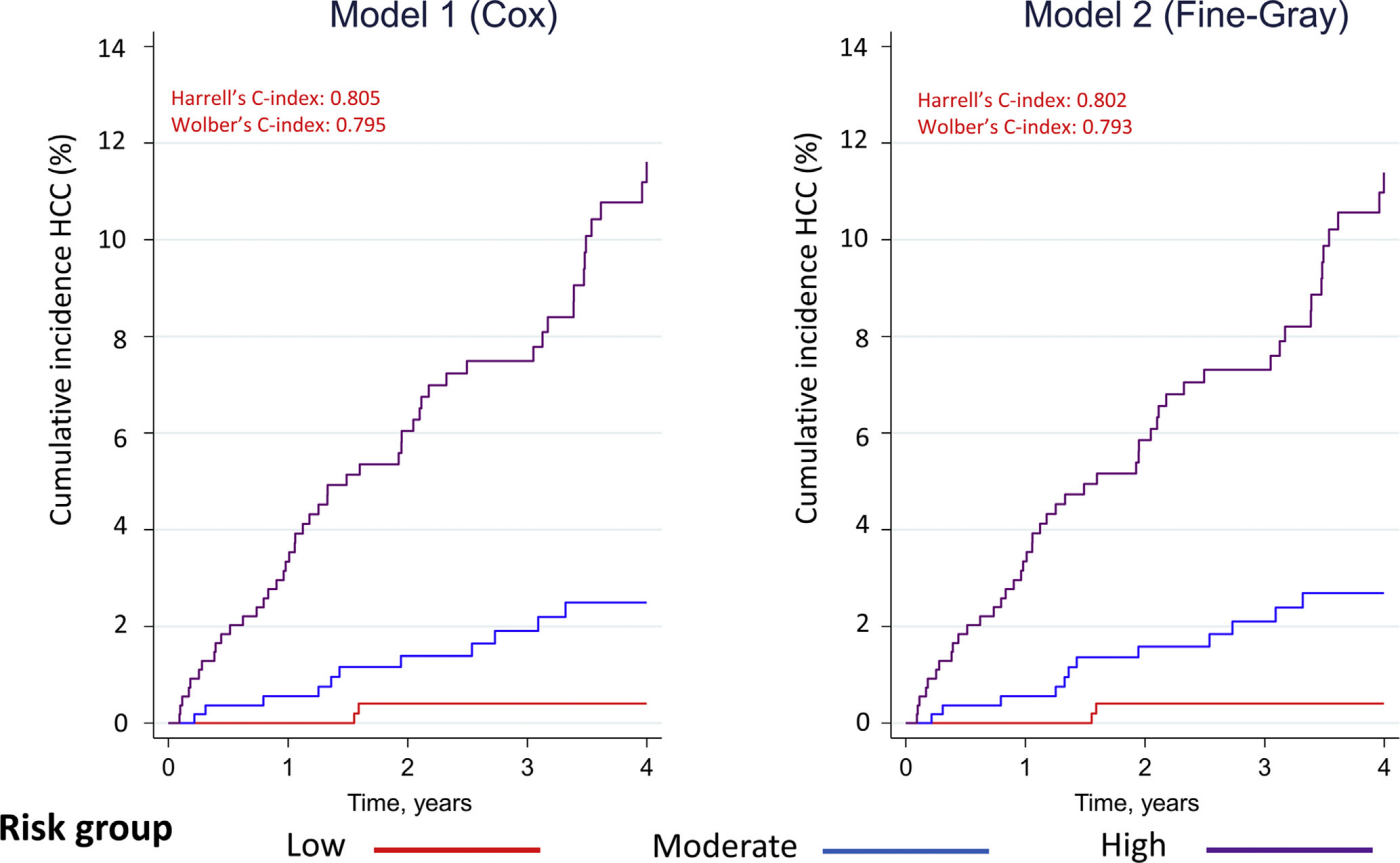
Cumulative incidence of HCC as per high, moderate, and low risk: model 1 vs model 2. The separation in HCC cumulative incidence between the 3 risk groups is similar for model 1 and model 2. This is consistent with both models having comparable levels of discrimination. High risk was defined as a risk score in the 67th percentile or greater (ie, top tertile); low risk was defined as a risk score in the 33rd percentile or lower (ie, bottom tertile). Moderate-risk patients are those whose risk score was in the middle tertile.

The discriminative ability of model 1 and model 2 was similar. Figure 1 shows equal separation in the cumulative incidence of HCC between risk tertiles for model 1 and 2.

Harrell’s c-index was 0.805 (95% CI: 0.761–0.849) for model 1 and 0.802 (95% CI: 0.757–0.847) for model 2. The Wolbers-modified C-index was 0.795 (95% CI: 0.751–0.838) for model 1 and 0.793 (95% CI: 0.749–0.837) for model 2.

There was also close agreement (ie, 97.2%) between high-risk patients identified in model 1 vs model 2 (Table A5). The same was true with respect to agreement for low-risk patients. This close agreement remained apparent when alternative definitions of high risk were considered (Tables A6 and A7).

### Three-year HCC Probability Agreement

**Figure 2:**
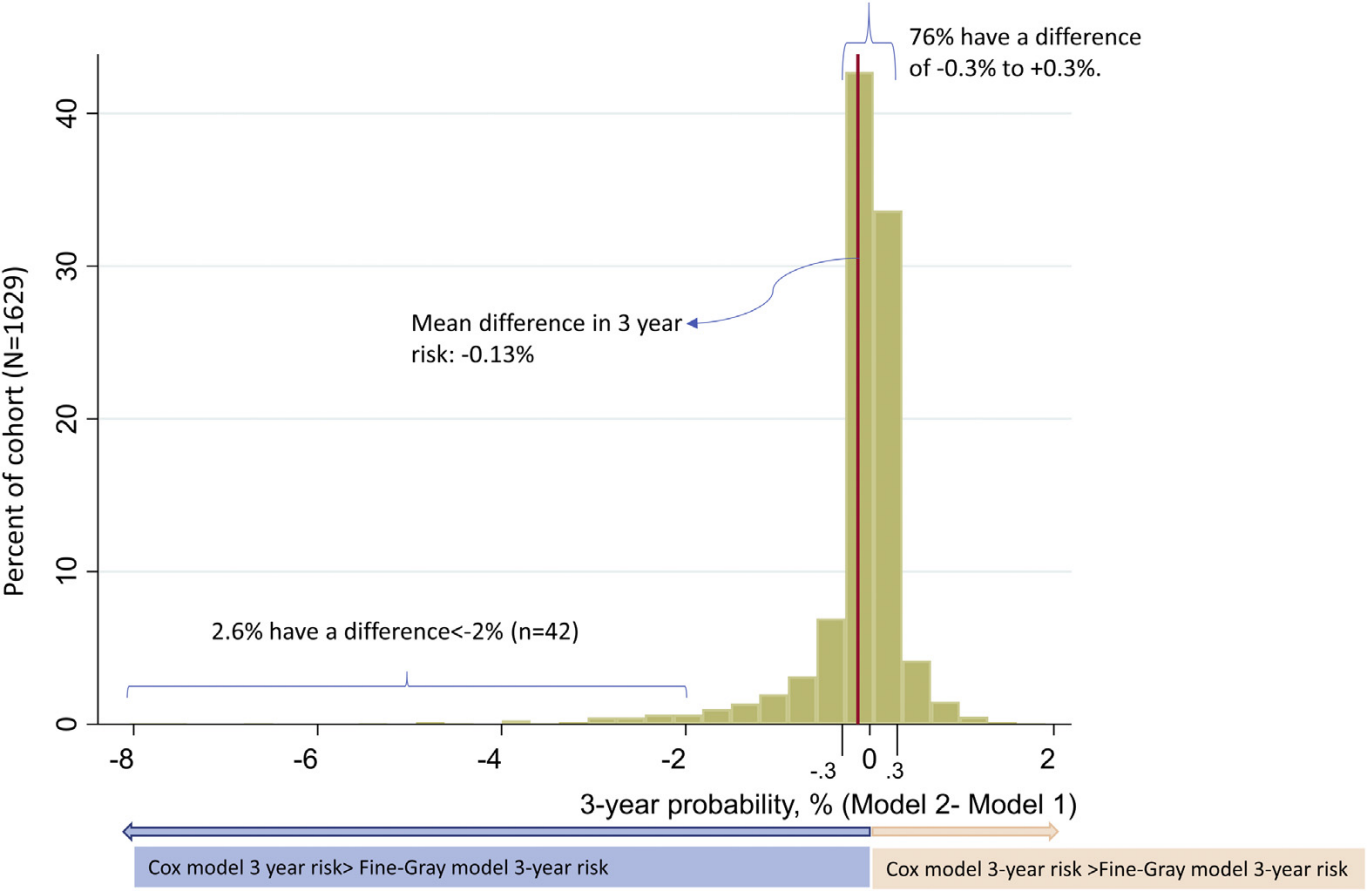
The histogram of 3-year predicted HCC risk (model 2 minus model 1).

**Figure 3:**
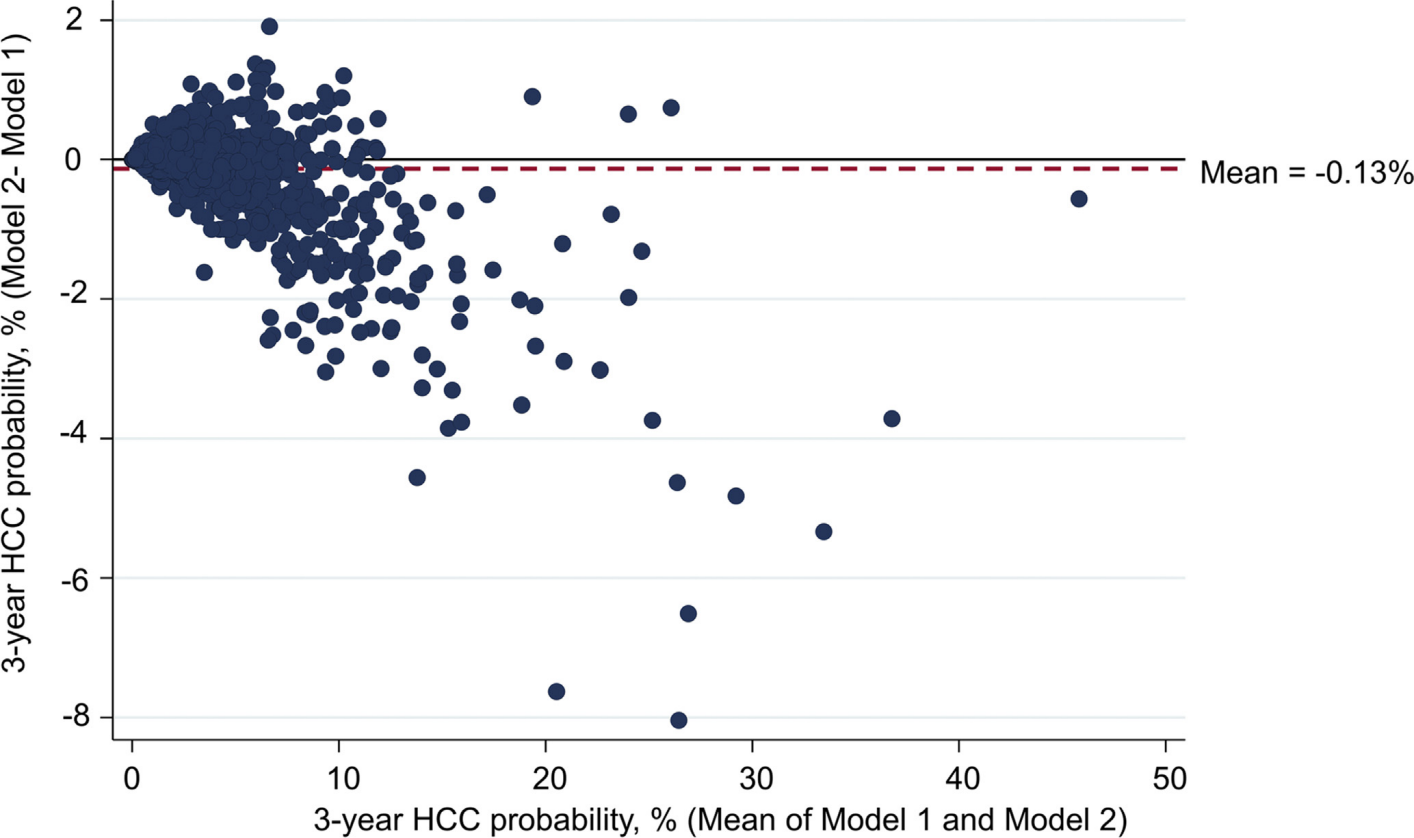
The scatter plot (Bland-Altman plot) of model 1 minus model 2 predicted 3-year HCC probability (vertical axis) and mean of model 1 and model 2 HCC probability (horizontal axis). There are 1629 circles in this scatter plot, one for each participant in the sample. The horizontal black line denotes the point at which model 1 and model 2 predictions are equal. Points below the black line indicate the Cox model (model 1) prediction is greater than the Fine-Gray model (model 2). In addition, vice versa, points above the black line indicate Cox model (model 1) prediction is less than the Fine-Gray model (model 2). The dashed red line is the mean value for the model 2 prediction minus the model 1 prediction.

Three-year predicted risks from model 1 were higher on average than for model 2 (Figures 2 and 3). The mean predicted 3-year HCC probability was 3.37% for model 1 vs 3.24% for model 2. Accordingly, the average difference was 0.13%. The median predicted 3-year HCC probability was considerably lower at 2.00% for model 1 vs 2.05% for model 2.

For most individuals, there was very close agreement between 3-year predicted risk in model 1 and model 2. Specifically, more than 3-quarters (76%) had a difference in 3-year predicted probability that was between −0.3% and +0.3% (Figures 2 and 3).

However, model 1 and model 2 predictions were discordant in some cases. Overall, 6.7% (110/1629) had a difference in 3-year probability exceeding 1%, and 2.6% (43/1629) had a difference exceeding 2% (Figure 2).

**Figure 4:**
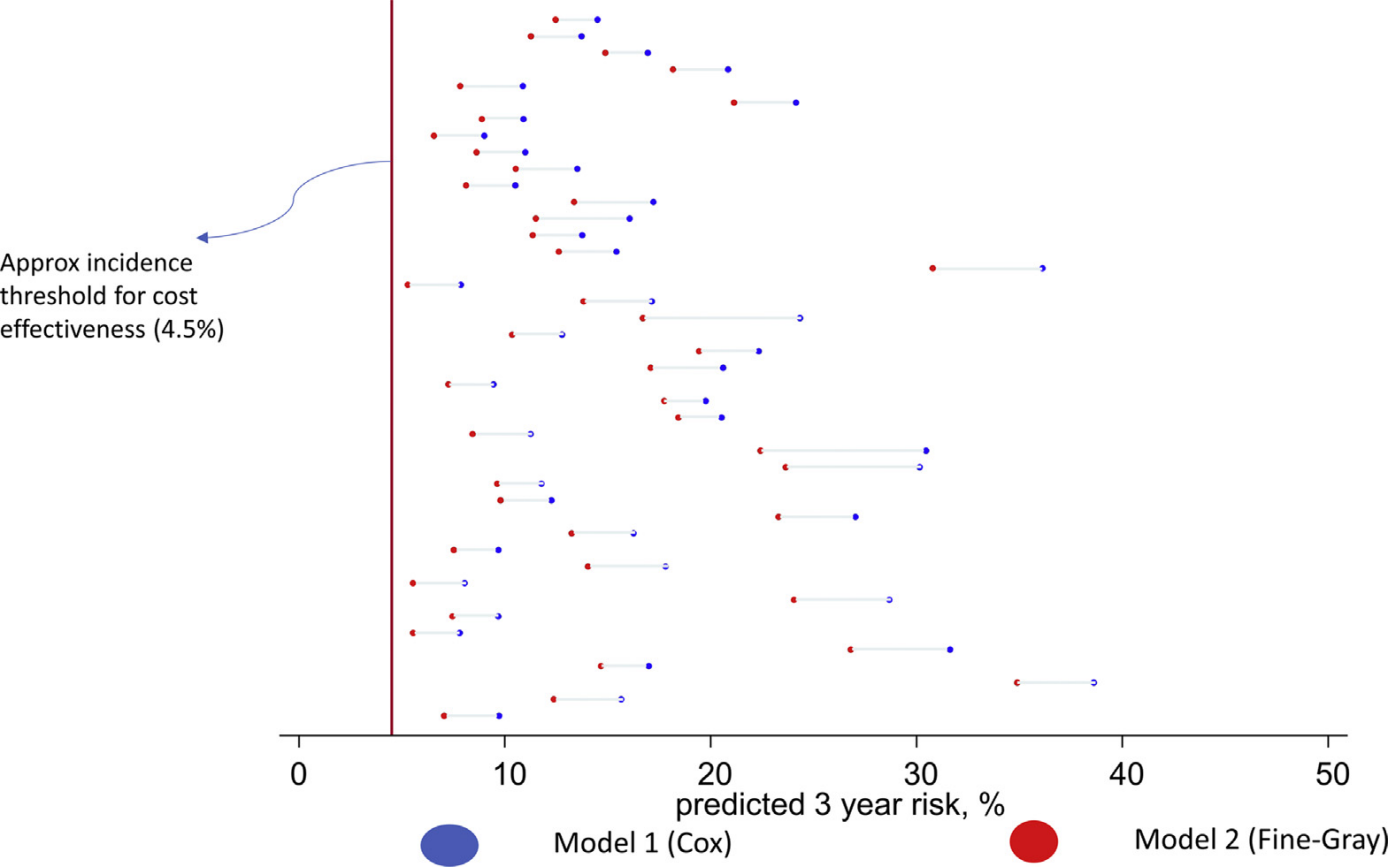
Three-year predicted HCC probability for patients where there is at least a 2% absolute difference between model 1 and model 2 prediction (N=43). There are 43 rows in this graph; each row indicates the 3-year risk of HCC predicted by model 1 (blue dot) and the 3-year risk predicted by model 2 (red dot) for a specific patient. Only patients with >2% absolute difference in 3-year risk predicted by model 1 and model 2 are shown in this plot.

The Bland-Altman plot shows a clear tendency for agreement to decline as HCC risk increases (Figure 3). Indeed, the largest differences (ie, those exceeding 2%) were all apparent in individuals with an HCC probability of at least 5% in both models (Figure 4 and Figure A2).

## Discussion

### Principal Findings

This is the first study to quantify the impact of competing risk bias in a HCC prediction modeling context. We developed 2 models predicting HCC occurrence that were identical in all respects except for the fact that model 1 ignored non-HCC mortality as a competing risk event, whereas model 2 did not. On the whole, we found that the 2 models generated very similar predictions. For example, the mean predicted 3-year probability of HCC was 3.37% for model 1 vs 3.24% for model 2. Thus, the average difference was only 0.13% (ie, 0.043% per year). Moreover, for the vast majority of patients (76%), the 3-year probability of HCC predicted by model 1 and model 2 was within ±0.3% of each other. In addition, the two models exhibited similar levels of discrimination and more or less picked out the same set of patients as “low” or “high” risk.

On the other hand, there were a minority of patients who did exhibit a considerably lower 3-year probability with model 2 vs model 1 (ie, at least a 1% difference). However, these were mostly patients whose 3-year HCC risk exceeded 5% in both models, which is well above the threshold at which clinicians would typically offer screening.^29^^,^^30^ Thus, although a competing risk model would lead to a more accurate prediction in these patients, it would probably not affect clinical decision-making. That said, patient counseling and clinical “buy in” hinge on the predicted risk of HCC occurrence being as accurate as possible. Because a competing risk perspective is relatively straightforward to incorporate into a prognostic modeling framework, we see no reason why future models predicting HCC occurrence should not adopt a competing risk perspective.

### Consistency With Previous Research

It is widely known that Cox regression generates inflated absolute risk predictions if competing risks are present. The level of inflation depends on 2 key factors: (a) the frequency of the competing risk event (the more frequent, the greater the bias) and (b) the length of the prediction time horizon (the longer the prediction horizon, the greater the bias).^31^ A study by Wolbers et al^26^ was one of the first to highlight the potential for competing risk bias to alter clinical decision-making. When predicting coronary heart disease (CHD) in women aged 55–90 years, Wolbers et al found that a naïve cox regression analysis classified 18% of individuals as “high risk” compared with only 8% when competing risks were accounted for. However, this could be seen as an extreme example insofar as more than a quarter of the population experienced the competing risk event, and the prediction horizon was quite long at 10 years. In contrast, analogous studies for other prognostic models with shorter prediction horizons and where the competing risk event in question is less frequent indicate a much more modest level of bias that would not alter clinical decision-making.^32^^,^^33^ Another very recent study by Cooper et al^34^ quantified competing risk bias for CHD predictions among individuals aged >65 years in New Zealand. They reported that the 5-year CHD risk was overestimated by ∼1% overall by the Cox model. Like our own study, they found that the degree of overestimation was much greater in the highest-risk patients (ie, 5%–6% in highest-risk patients vs ∼1% overall).

Taken together, previous studies demonstrate that the degree of competing risk bias is context-specific. This is why an empirical approach is needed to investigate prevalent concerns within the clinical community^20^ regarding the impact of competing risk bias on the validity of models predicting HCC occurrence.

### Strengths and Limitations

Our study has some limitations that warrant discussion. First, we included patients with cirrhosis and cured hepatitis C, a rapidly growing patient group where the need for effective risk-stratification tools is arguably at its most pronounced. It should be pointed out that our findings may not necessarily be generalizable to other cirrhosis etiologies. However, the frequency of competing risk events among patients with HCV cirrhosis is likely to surpass that of other cirrhosis etiologies (ie, due to the high frequency of alcohol use, drug use, and other health risk behaviors^35^, ^36^, ^37^). From this perspective, therefore, one would expect the degree of competing risk bias to be at its upper limit for patients with cirrhosis SVR—that is, less considerable for other cirrhosis etiologies. This would only further support our conclusion regarding the modest level of competing risk bias in HCC prognostic models. Second, we only explored the impact of competing risk bias in relation to a single time point (ie, 3 years after achieving SVR). As already discussed, it is likely that the impact of ignoring competing risks would be more substantial if a longer time horizon was considered. However, a 3-year time horizon is probably most relevant for making HCC screening decisions, and it also aligns with time horizon emphasized by previous prediction models for HCC.^12^ Third, it is very difficult to define what degree of bias is clinically relevant. In this study, we focused simply on reporting the proportion of patients for whom the 3-year HCC probability differed by more than ±1% and by more than ±2%. However, it is possible that differences that are smaller than this could still be clinically relevant. Fourth, the association between reported gender and HCC was imprecise because only 7 of 82 HCC cases were female. Finally, we were missing data on some important prognostic factors—particularly GGT and liver stiffness which have been used in 2 previous models. However, the omission of these 2 variables is unlikely to have influenced our conclusions.

## Conclusion

In conclusion, we highlight the importance of adopting a competing risk perspective when developing prognostic models for HCC occurrence. However, our data also reassure the clinical community that the competing risk bias inherent in existing HCC occurrence models is unlikely to affect clinical decision-making in a serious way.

## Supplementary Materials

Figures A1, A2 and Tables A1–A7
