# Supplementary figures and images for: Competing Risk Bias in Prognostic Models Predicting Hepatocellular Carcinoma Occurrence: Impact on Clinical Decision-making
Source: Gastro Hep Adv. 2022 Feb 3;1(2):129–36. doi: 10.1016/j.gastha.2021.11.008 (PMC11307513; doi:10.1016/j.gastha.2021.11.008)

**SUPPLEMENTARY MATERIAL:**

**
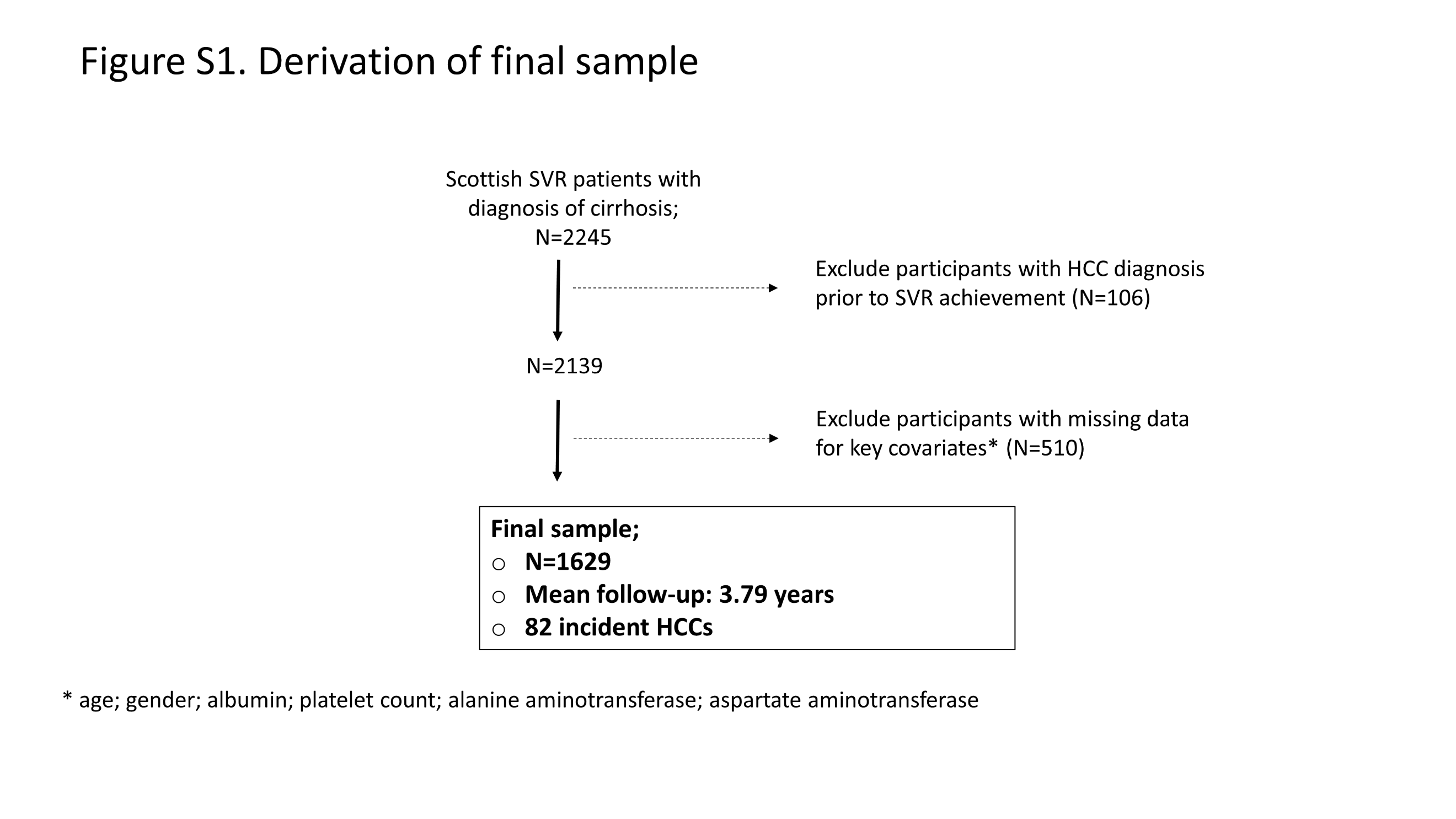
**

**
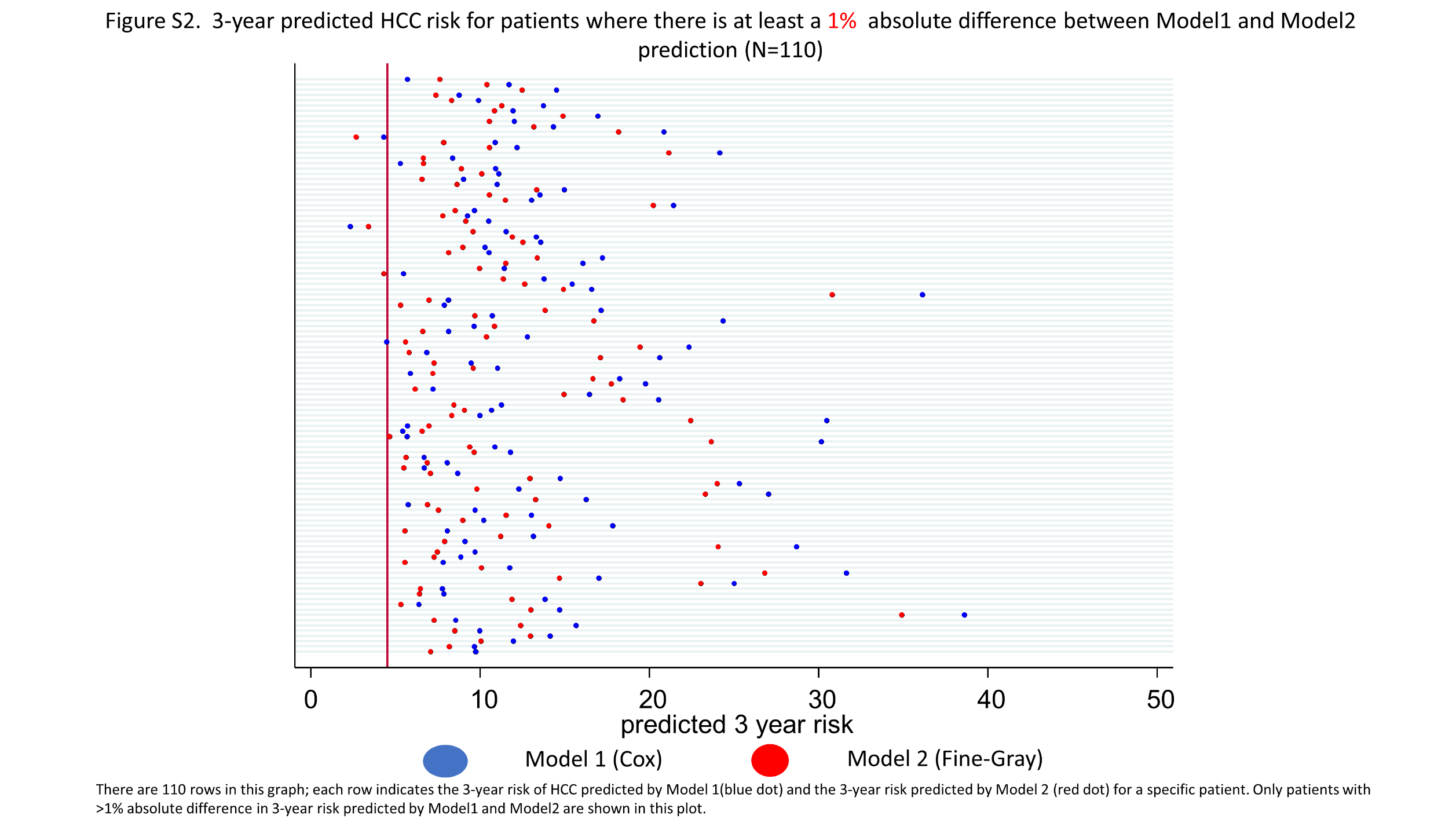
**

Supplement: Figures A1, A2 and Tables A1–A7 [file mmc1.docx]
